# The Impact of Admission Viral Load and Inflammatory Markers on Mortality and Prognosis in Crimean‐Congo Hemorrhagic Fever

**DOI:** 10.1002/jmv.71042

**Published:** 2026-07-04

**Authors:** Yasemin Çakır Kıymaz, Ertuğrul Keskin, Caner Öksüz, Seyit Ali Büyüktuna, Nazif Elaldı

**Affiliations:** ^1^ Department of Infectious Diseases and Clinical Microbiology Sivas Cumhuriyet University Sivas Turkey

**Keywords:** Crimean‐Congo hemorrhagic fever, CRP, LDH, mortality, prognosis, viral load

## Abstract

This study aimed to evaluate the impact of admission viral load and biochemical parameters on mortality and disease severity in a large cohort of patients with Crimean‐Congo Hemorrhagic Fever (CCHF). This retrospective cohort study included 492 adult patients diagnosed with CCHF between 2022 and 2025. Admission viral loads, laboratory parameters, and clinical findings were compared. Logistic regression and ROC analyses were performed to identify independent predictors and optimal cut‐off values. The mortality rate was 9.8% (*n* = 48). In multivariate analysis, admission log‐viral load was identified as a potent independent predictor of mortality (OR: 3.36, *p* < 0.001 95% CI: 2.88–8.95, *p* < 0.001), alongside CRP (OR: 1.02, *p* < 0.001). Notably, advanced age was a significant risk factor, associated with a 2.8% increased risk of mortality (OR: 1.028, *p* = 0.025). In a separate model for disease severity, log‐viral load (OR: 1.71, *p* < 0.001), age (OR: 1.021, *p* = 0.002), and CRP (OR: 1.012, *p* < 0.001) were significant independent predictors of a severe clinical course. Conversely, higher fibrinogen levels (OR: 0.997, *p* = 0.025) were associated with reduced disease severity. A viral load cut‐off of 6.69 log_10_ copies/mL predicted mortality with 85.4% sensitivity and a negative predictive value of 98%. Admission viral load serves as a primary early marker for both mortality and clinical severity in CCHF. While CRP reflects the host's inflammatory response, admission fibrinogen levels also provide critical prognostic information. Combined use of these markers can optimize early triage and clinical management in endemic regions.

## 1 Introduction

Crimean‐Congo hemorrhagic fever (CCHF) is a zoonotic infectious disease with a case fatality rate reaching up to 40% and a broad, expanding geographical distribution [1]. CCHF is primarily prevalent in agricultural and livestock‐breeding communities; it is transmitted to humans via infected tick bites, the crushing of engorged ticks, or through direct contact with the infected blood or tissues of viremic animals [2]. The disease exhibits a wide clinical spectrum, ranging from asymptomatic infections to fatal severe cases [3]. While the clinical course is subclinical or self‐limiting in many instances, symptomatic patients show significant clinical heterogeneity. The disease may manifest initially with non‐specific prodromal symptoms such as fever, headache, and myalgia; however, it can progress to life‐threatening multiorgan failure characterized by major hemorrhages, hepatic dysfunction, neurological disturbances, and hemodynamic instability [4]. This unpredictability in the clinical course of CCHF complicates the early‐stage prediction of prognosis.

Numerous studies in the literature have aimed to identify prognostic factors, generally focusing on hematological/biochemical parameters, severity scoring systems, and novel biomarkers [5, 6, 7]. Thrombocytopenia, elevated liver enzymes, and prolonged coagulation times are the most frequently reported conventional parameters for predicting clinical outcomes [8]. Although studies investigating the relationship between viral load and mortality exist, they have often been limited by small sample sizes and have not sufficiently validated the potential of initial viral load levels to predict disease severity [9, 10]. Consequently, large‐scale studies are required to elucidate the decisive role of viral load for a better understanding of CCHF prognosis. Leveraging a larger patient cohort than many existing studies, our research aims to evaluate the relationship between initial viral load and clinical outcomes on a more robust statistical foundation. The primary objective is to determine the role of viral load in disease severity and mortality, thereby contributing to the optimization of clinical management strategies.

## 2 Materials and Methods

### 2.1 Study Population

This study was designed as a retrospective cohort analysis. A total of 492 adult patients diagnosed with CCHF and followed at the Department of Infectious Diseases and Clinical Microbiology, Sivas Cumhuriyet University Hospital, between April 2022 and August 2025 were included. CCHF diagnosis was confirmed via polymerase chain reaction (PCR). Patients with “probable” diagnosis but lacking microbiological confirmation, those with missing admission viral load data, and patients under the age of 18 were excluded. Demographic characteristics, clinical findings, and laboratory parameters were obtained from hospital registry systems and patient files. Patients were categorized into two groups for comparison: survivors versus non‐survivors, and mild versus moderate‐severe cases. Clinical findings, laboratory parameters, and viral load values at admission were compared between these groups. Disease severity was classified using the Severity Grading Score (SGS) [8].

### 2.2 Statistical Analysis

Statistical analyses were performed using SPSS (Version 23.0; IBM Corp., Armonk, NY, USA), and data visualization was conducted using GraphPad Prism (Version 8.3.0). The distribution of continuous variables was assessed using the Kolmogorov–Smirnov test. Normally distributed variables were expressed as mean ± standard deviation, while non‐normally distributed variables were presented as median (1st–3rd quartiles). Categorical variables were presented as numbers and percentages. For intergroup comparisons, the Independent Samples *t*‐test was used for normally distributed data, the Mann–Whitney U test for non‐normally distributed data, and Chi‐square or Fisher's exact tests for categorical variables.

The performance of viral and laboratory parameters in predicting mortality and prognosis was evaluated using ROC analysis to determine optimal cut‐off values. Logistic regression analysis was employed to identify independent factors associated with severe disease. Due to the limited number of fatal cases (I = 48), the number of variables in the multivariate model was restricted to prevent overfitting. Parameters found significant in univariate analysis were stepwise included in the model based on their clinical significance in the literature and statistical significance levels. A *p*‐value of < 0.05 was considered statistically significant.

### 2.3 Ethical Approval

This study was approved by the Sivas Cumhuriyet University Social Sciences and Humanities Ethics Committee (Decision no: 2025‐09/05, Date: 18.09.2025) and was conducted in accordance with the principles of the Declaration of Helsinki (Table 1).

**Table 1:** Comparison of demographic and clinical characteristics between survivors and non‐survivors with CCHF.

Variables	Total (*n* = 492)	Survivor (*n* = 444)	Non‐survivor (*n* = 48)	*p* value
Demographic characteristics				
Age (years)	52 (32–64)	52 (37–64)	62 (36–74)	**0.03**
Gender				0.95
Male	340 (69.1)	307 (69.1)	33 (68.8)	
Female	152 (30.9)	137 (30.9)	15 (31.3)	
Area of residence				0.95
Urban	340 (69.1)	307 (69.1)	33 (68.8)	
Rural	152 (30.9)	137 (30.9)	15 (31.3)	
Occupation				0.42
Agriculture/Animal husbandry	388 (78.9)	348 (78.4)	40 (83.3)	
Others	104 (21.1)	96 (21.6)	8 (16.7)	
Yes	358 (72.8)	335 (75.5)	23 (47.9)
No	134 (27.2)	109 (24.5)	25 (52.1)
History of contact with animals				0.43
Yes	150 (30.5)	133 (30.0)	17 (35.4)	
No	342 (69.5)	311 (70.0)	31 (64.6)	
History of contact with a CCHF patient				0.06
Yes	24 (4.9)	19 (4.3)	5 (10.4)	
No	468 (95.1)	425 (95.7)	43 (89.6)	
Clinical Features				
Malaise/Fatigue	461 (93.7)	414 (93.2)	47 (97.9)	0.34
Myalgia	453 (92.1)	407 (91.7)	46 (95.8)	0.40
Fever	382 (77.6)	349 (78.6)	33 (68.8)	0.12
Headache	336 (68.3)	309 (69.6)	27 (56.3)	0.05
Nausea/Vomiting	215 (43.7)	189 (42.6)	26 (54.2)	0.12
Diarrhea	142 (28.9)	118 (26.6)	24 (50)	**< 0.001**
Altered mental status	10 (2.0)	6 (1.4)	4 (8.3)	**0.01**
Time from symptom onset to admission (days)	4 (2–5)	4 (2–5.7)	4 (2–5)	0.83
Hemorrhagic manifestations				
At admission	38 (7.7)	27 (6.1)	11 (22.9)	**< 0.001**
Developed during hospitalization	128 (26)	83 (18.7)	45 (93.8)	< **0.001**
Sites of hemorrhage				
Petechiae/Purpura/Ecchymosis	71 (14.4)	56 (12.6)	15 (31.3)	**< 0.001**
Mucosal hemorrhage (Epistaxis, Gingival, etc.)	67 (13.6)	48 (10.8)	19 (39.6)	< **0.001**
Genitourinary hemorrhage	31 (6.3)	20 (4.5)	11 (22.9)	< **0.001**
Gastrointestinal hemorrhage	23 (4.7)	13 (2.9)	10 (20.8)	< **0.001**
Pulmonary hemorrhage	19 (3.9)	10 (2.3)	9 (18.8)	< **0.001**
Intracranial hemorrhage	4 (0.8)	1 (0.2)	3 (6.3)	< **0.01**
SGS on admission	2 (1–4)	2 (1–3)	5 (4–8)	**< 0.001**
Disease severity				**< 0.001**
Mild	386 (78.5)	374 (84.2)	12 (25)	
Moderate‐Severe	106 (21.5)	70 (15.8)	36 (75)	
Hospital course and outcomes				
Length of hospital stay (days)	6 (4–8)	6 (4–8)	5 (3–7)	**< 0.01**
Admission to ICU	56 (11.4)	10 (2.3)	46 (95.8)	**< 0.001**

*Note:* Numerical parameters following a normal distribution were compared using an independent t‐test. Non‐normally distributed numerical data were compared using the Mann‐Whitney U test. Categorical variables are presented as n (%), normally distributed numerical data as mean ± SD, and non‐normally distributed numerical data as median (1st‐3rd quartiles). *p* < 0.05 values are shown in bold. *p* values < 0.05 were considered statistically significant.

## 3 Results

### 3.1 Demographic and Clinical Characteristics

Of the 492 CCHF patients included, 90.2% (*n* = 444) were discharged after recovery, while 9.8% (*n* = 48) resulted in mortality. In group comparisons, non‐survivors were significantly older (median 62 vs. 52 years, *p* = 0.03). Among clinical symptoms, diarrhea (50% vs. 26.6%, *p* < 0.001) and altered consciousness (8.3% vs. 1.4%, *p* = 0.01) were more common in the mortality group. Furthermore, hemorrhagic manifestations during hospitalization (93.8% vs. 18.7%), particularly gastrointestinal, pulmonary, and mucosal hemorrhages were dramatically higher in the non‐survivor group (*p* < 0.001) (Table 2).

**Table 2:** Comparison of baseline viral load and laboratory parameters in survivors and non‐survivors.

Variables	Reference range	Survivors (*n* = 444)	Non‐survivors (*n* = 48)	*p* value
Hematological parameters				
WBC (10^9^/L)	4.0–10.0	2.83 (2.0–3.8)	4.61 (2.3–6.6)	0.10
HGB (g/dL)	12.0–16.0*	14.2 (12.9–15.1)	13.3 (11.6–14.7)	0.09
PLT (10^9^/L)	150–400	98 (64–131)	47 (25–78)	**< 0.001**
Biochemical parameters				
AST (U/L)	< 40	78 (38–163)	273 (145–778)	**< 0.001**
ALT (U/L)	< 40	46 (24–92)	81 (45–310)	**< 0.001**
BUN (mg/dL)	7–20	15.2 (11.6–18.9)	21 (17.3–40)	**< 0.001**
Creatinine (mg/dL)	0.5–1.2	0.89 (0.73–1.0)	1.1 (0.9–1.9)	**< 0.001**
LDH (U/L)	140–280	354 (260–504)	875 (630–1741)	**< 0.001**
CPK (U/L)	< 200	229 (156–282)	328 (183–753)	**< 0.001**
CRP (mg/L)	< 5	11.8 (4.8–30.1)	42.9 (18.5–102)	**< 0.001**
Coagulation Parameters				
PT (s)	11.0–13.5	10.5 (8.9–12)	13.6 (11–18.3)	**< 0.001**
aPTT (s)	25.0–35.0	29.1 (26.1–33.1)	42.8 (35–56.1)	**< 0.001**
INR	0.8–1.2	1.0 (0.9–1.1)	1.3 (1.1–1.7)	**< 0.001**
Fibrinogen (mg/dL)	200–400	272 (235–317)	238 (156–282)	**0.01**
d‐dimer (mg/L)	< 0.5	1.6 (0.8–4.4)	15.7 (3.9–35)	**< 0.001**
Virological Parameters				
Viral load (log_10_ copies/mL)	—	5.84 (4.44–6.85)	7.43 (6.85–8.34)	**< 0.001**

*Note:* Numerical parameters following a normal distribution were compared using an independent *t*‐test. Non‐normally distributed numerical data were compared using the Mann–Whitney U test. Categorical variables are presented as n (%), normally distributed numerical data as mean ± SD, and non‐normally distributed numerical data as median (1st‐3rd quartiles). *p* < 0.05 values are shown in bold. *p* values < 0.05 were considered statistically significant.

Abbreviations: aPTT, Activated partial thromboplastin time; ALT, Alanine aminotransferase; AST, Aspartate aminotransferase; BUN, Blood urea nitrogen; CPK, Creatin kinase; CRP; C reactive protein; HGB, Hemoglobin; INR, International normalized ratio; LDH, Lactate dehydrogenases; PT, Prothrombin time; PLT, Platelets; RBC, Red blood cells; WBC, White blood cells.

### 3.2 Laboratory Findings and Viral Load

Initial laboratory parameters revealed significantly lower platelet counts (47 vs. 98 × 10^9^/L, *p* < 0.001) and significantly higher levels of liver enzymes (AST, ALT), LDH, CPK, CRP, BUN, and Creatinine in the non‐survivor group (*p* < 0.001). Regarding coagulation profiles, PT, aPTT, INR, and d‐dimer levels were markedly prolonged/elevated in non‐survivors, while fibrinogen levels were significantly lower (*p* = 0.01). A similar laboratory profile was observed when comparing “moderate‐severe” cases with “mild” cases (Table 3). Admission viral load was significantly higher in non‐survivors (7.43 log_10_ copies/mL) than in survivors (5.84 log_10_ copies/mL) (*p* < 0.001).

**Table 3:** Comparison of baseline viral load and laboratory parameters in mild and moderate‐severe disease.

Variables	Reference range	Mild (*n* = 386)	Moderate‐severe (*n* = 106)	*p* value
Hematological Parameters				
WBC (10^9^/L)	4.0–10.0	2.8 (2.1–3.7)	2.8 (1.8–4.7)	0.19
HGB (g/dL)	12.0–16.0*	15.2 (14.3–16.2)	15 (13.9–16.2)	0.06
PLT (10^9^/L)	150–400	107 (84–136)	48 (26–89)	**< 0.001**
Biochemical parameters				
AST (U/L)	< 40	63 (33–105)	219 (92–418)	**< 0.001**
ALT (U/L)	< 40	38 (22–67)	90 (42–181)	**< 0.001**
LDH (U/L)	140–280	322 (247–417)	649 (378–897)	**< 0.001**
CPK (U/L)	< 200	205 (112–444)	379 (189–1017)	**< 0.001**
BUN (mg/dL)	7–20	15.1 (11.6–18.1)	17.1 (12.9–23.9)	**< 0.001**
Creatinine (mg/dL)	0.5–1.2	0.8 (0.7–1.0)	0.9 (0.8–1.1)	**< 0.001**
CRP (mg/L)	< 5	10.2 (4.2–24)	25.9 (7.8–54.9)	**< 0.001**
Coagulation parameters				
PT (s)	11.0–13.5	10.4 (8.8–11.7)	11.3 (9.6–13.5)	**< 0.001**
aPTT (s)	25.0–35.0	28.3 (25.7–31.8)	34.6 (28.9–42.7)	**< 0.001**
INR	0.8–1.2	1.0 (0.9–1.1)	1.0 (0.9–1.2)	**< 0.001**
Fibrinogen (mg/dL)	200–400	281 (244–319)	249 (208–285)	**< 0.001**
d‐dimer (mg/L)	< 0.5	1.3 (0.7–3.3)	4.3 (1.3–20.6)	**< 0.001**
Virological parameters				
Viral load (log_10_ copies/mL)	—	5.55 (4.18–6.46)	6.97 (5.88–7.63)	**< 0.001**

*Note:* Numerical parameters following a normal distribution were compared using an independent t‐test. Non‐normally distributed numerical data were compared using the Mann–Whitney U test. Categorical variables are presented as n (%), normally distributed numerical data as mean ± SD, and non‐normally distributed numerical data as median (1st‐3rd quartiles). *p* < 0.05 values are shown in bold. *p* values < 0.05 were considered statistically significant.

Abbreviations: aPTT, Activated partial thromboplastin time; ALT, Alanine aminotransferase; AST, Aspartate aminotransferase; BUN, Blood urea nitrogen; CRP, C reactive protein; CPK, Creatin kinase; HGB, Hemoglobin; INR, International normalized ratio; LDH, Lactate dehydrogenases; PLT, Platelets; PT, Prothrombin time; RBC, Red blood cells; WBC, White blood cells.

### 3.3 Predictive Factors for Mortality and Disease Severity (ROC Analysis)

ROC analysis indicated that most biochemical, hematological, and virological variables had high discriminatory power for mortality. LDH (AUC: 0.894), aPTT (AUC: 0.870), d‐dimer (AUC: 0.869), and INR (AUC: 0.861) emerged as parameters with the highest diagnostic accuracy (Table 4). A viral load above 6.69 log_10_ copies/mL predicted mortality with 85.4% sensitivity and 74.1% specificity. For predicting disease severity (severe course), LDH (AUC: 0.965), AST (AUC: 0.962), and low platelet count (AUC: 0.949) were excellent markers (Table 5). Notably, INR was the most specific parameter for predicting mortality, with 94.8% specificity and a positive likelihood ratio (LR + ) of 12.8. Regarding organ function, AST (> 191 U/L; AUC: 0.840) and BUN (> 19.7 mg/dL; AUC: 0.830) showed higher diagnostic accuracy than ALT and Creatinine. Conversely, Fibrinogen (AUC: 0.633) and CPK (AUC: 0.666) demonstrated limited performance. All parameters exhibited high negative predictive values (NPV) (93.6% to 98.4%), confirming that values below the determined cut‐offs are strong clinical indicators of survival.

**Table 4:** ROC analysis results for variables in predicting mortality.

	Cut‐off	AUC	Sen%	Spe (%)	LR +	LR‐	PPV	NPV
Viral load (log_10_ copies/mL)	> 6.69	0.847	85.4	74.1	3.3	0.2	26.3	98
PLT (10^9^/L)	≤ 54	0.798	75	81	3.9	0.3	29.8	97
AST (U/L)	> 191	0.840	79.1	78.6	3.7	0.2	28.6	97.2
ALT (U/L)	> 68	0.762	70.8	67.7	2.2	0.4	19.2	95.6
BUN (mg/dL)	> 19.7	0.830	72.9	77.2	3.2	0.3	25.7	96.3
Creatinine (mg/dL)	> 1.22	0.778	54.1	90.3	5.5	0.5	37.7	94.8
CRP (mg/L)	> 18	0.810	85.4	62.2	2.2	0.2	20	97.5
LDH (U/L)	> 600	0.894	87.5	82.4	4.9	0.1	35	98.4
CPK (U/L)	> 279	0.666	68.7	56.5	1.5	0.5	14.6	94.4
PT (s)	> 12.8	0.827	70.8	87.6	5.7	0.3	38.2	96.5
aPTT (s)	> 39.4	0.870	75	90.9	8.3	0.2	47.7	97.1
INR	> 1.35	0.861	66.6	94.8	12.8	0.3	58.2	96.3
Fibrinogen (mg/dL)	≤ 217	0.633	45.8	85.3	3.1	0.6	25.3	93.6
d‐dimer (mg/L)	> 5.01	0.869	85.4	77.2	3.7	0.1	28.9	98

Abbreviations: aPTT, Activated partial thromboplastin time; ALT, Alanine aminotransferase; AST, Aspartate aminotransferase; BUN, Blood urea nitrogen; CRP; C reactive protein; CPK, Creatin kinase; HGB, Hemoglobin; INR, International normalized ratio; LDH, Lactate dehydrogenases; PLT, Platelets; PT, Prothrombin time; RBC, Red blood cells; WBC, White blood cells.

**Table 5:** ROC analysis results for variables in predicting severe disease.

	Cut‐off	AUC	Sen%	Spe (%)	LR +	LR‐	PPV	NPV
Viral load (log_10_ copies/mL)	> 5.6	0.586	72.6	44.3	1.3	0.6	26.4	85.5
PLT (10^9^/L)	≤ 56	0.949	86.7	91.4	10.1	0.14	73.6	96.2
AST	> 161	0.962	95.2	86.5	7.0	0.05	66.0	98.5
ALT	> 68	0.895	85.7	77.7	3.8	0.1	51.1	95.2
BUN	> 18.3	0.635	51.8	73.3	1.9	0.6	34.8	84.7
Creatinine	> 1.25	0.623	30.1	91.7	3.6	0.7	50.0	82.7
CRP	> 28.3	0.604	45.1	72.3	1.6	0.7	30.5	83.0
LDH	> 583	0.965	88.5	92.4	11.7	0.1	76.2	96.7
CPK	> 232	0.780	85.7	57.6	2.0	0.2	35.6	93.7
PT	> 12.8	0.580	33.3	86.2	2.4	0.7	39.8	82.6
aPTT	> 35	0.830	69.2	89.1	6.3	0.3	63.2	91.5
INR	> 1.4	0.559	28.5	97.1	10.0	0.7	73.2	83.3
Fibrinogen	≤ 250.1	0.641	58.2	67.5	1.7	0.6	32.4	85.8
d‐dimer	> 5.6	0.642	43.9	81.0	2.3	0.6	36.0	85.6

Abbreviations: aPTT, activated partial thromboplastin time; ALT, alanine aminotransferase; AST, aspartate aminotransferase; BUN, blood urea nitrogen; CPK, Creatin kinase; CRP; C reactive protein; HGB, Hemoglobin; INR, International normalized ratio; LDH, Lactate dehydrogenases; PLT, Platelets; PT, Prothrombin time; RBC, Red blood cells; WBC, White blood cells.

### 3.4 Predictors of Mortality and Disease Severity (Regression Analysis)

Multivariate logistic regression analysis was performed to identify independent predictors of mortality (Table 6). The established model was statistically significant. According to the results, log‐viral load was identified as one of the most potent independent risk factors for mortality (*p* < 0.001). Each unit increase in log‐viral load was associated with a 3.36‐fold increase in the risk of death (95% CI: 2.15–5.23). Similarly, elevated CRP levels were significantly associated with mortality (*p* < 0.001); each unit increase in CRP resulted in a 2.0% increase in the risk of death (OR: 1.020). Notably, advanced age was a significant risk factor, associated with a 2.8% increased risk of mortality (OR: 1.028, *p* = 0.025). Fibrinogen level (*p* = 0.814) did not show a statistically significant independent effect on mortality in this model.

**Table 6:** Results of regression analyses for mortality.

Variables	Wald's x	OR (%95 Cl)	*p* value
Age (years)	5.017	1.028 (1.003–1.053)	0.025
Viral load (log_10_ copies/mL)	28.764	3.363 (2.159–5.238)	**< 0.001**
Fibrinogen (mg/dL)	0.056	1.000 (0.0999–1.001)	0.814
CRP (mg/L)	20.703	1.020 (1.011–1.029)	**< 0.001**

*Note:* Hosmer‐Lemeshow *p* = 0.689.

Abbreviations: Cl, Confidence interval; CRP, C‐reactive protein; OR, Odds ratio.

The independent risk factors affecting disease severity were evaluated in a separate multivariate logistic regression model (Table 7). In this model, age, log‐viral load, fibrinogen, and CRP, were found to be significant independent predictors of clinical severity (*p* < 0.05). Each unit increase in log‐viral load increased the probability of a severe clinical course 1.71‐fold (95% CI: 1.453–2.025; *p* < 0.001). Similarly, both age (OR: 1.021; *p* = 0.002 ) and CRP levels (OR: 1.012; *p* < 0.001) were significantly associated with increased disease severity. Conversely, an inverse relationship was observed between fibrinogen levels and disease severity (*B* = −0.003; *p* = 0.025 ); each unit increase in fibrinogen reduced the risk of severe disease by 0.3% (OR: 0.997).

**Table 7:** Results of regression analyses for severe disease.

Variables	Wald's *x*	OR (%95 Cl)	*p* value
Age (years)	9.328	1.021 (1.070–1.035)	0.00
Viral load (log_10_ copies/mL)	40.504	1.715 (1.453–2.025)	**< 0.001**
Fibrinogen (mg/dL)	5.037	0.997 (0.994–1.000)	**0.025**
CRP (mg/L)	13.263	1.012 (1.005–1.081)	**< 0.001**

*Note:* Hosmer‐Lemeshow *p* = 0.403.

Abbreviations: Cl, Confidence interval; CRP, C‐reactive protein; OR, Odds ratio; PLT, Platelets.

## 4 Discussion

Our study, conducted on a large cohort of 492 CCHF patients, clarifies the distinct roles of viral kinetics and host response in determining clinical outcomes. The most striking finding of our analysis is that while admission viral load is an independent predictor for both mortality and disease severity, its impact on survival is significantly more potent. Specifically, each unit increase in log‐viral load increases the risk of mortality 3.63‐fold, whereas it increases the risk of severe disease 1.71‐fold. This suggests that while viral replication initiates the pathogenic process across the entire clinical spectrum, it is the primary and most decisive driver of fatal outcomes.

In the present study, multivariate logistic regression identified log‐viral load as a potent independent predictor for both mortality and disease severity, emphasizing that active viral replication is the central element triggering CCHF pathogenesis. While previous studies reported similar associations, our data from a large population validate this relationship through multivariate analysis [9, 10, 11], supporting the use of admission viral load as a fundamental biomarker for early prognosis. Specifically, a viral load cut‐off of 6.69 log_10_ copies/mL predicted mortality with high sensitivity and a high negative predictive value (98%), facilitating effective resource management in endemic regions. However, a notable discrepancy was observed in discriminatory performance; while viral load accurately predicted mortality (AUC: 0.847), its performance in classifying disease severity was weak (AUC: 0.586). This suggests that while viral replication acts as the primary “trigger” or “ignition” for the pathogenic cascade, its role in determining clinical severity is less decisive than its role in mortality. Once the disease reaches the clinical stage, the progression and severity appear to be more heavily influenced by the host's inflammatory response represented by markers such as CRP rather than the viral burden alone.

In our analysis, age emerged as a pivotal factor in the clinical progression and outcome of CCHF. Multivariate analysis identified advanced age as a significant independent predictor for both disease severity and mortality. Specifically, each year of increase in age was associated with a 2.8% increase in mortality risk (OR: 1.028, *p* = 0.025) and a significant increase in the probability of a severe clinical course (OR: 1.021, *p* = 0.002). This suggests that advanced age consistently contributes to a more aggressive disease phenotype and a higher risk of fatal outcome, independent of viral load and inflammatory markers such as CRP. The impact of age is likely attributed to reduced physiological reserve, the presence of underlying comorbidities, and age‐related changes in the immune system often referred to as immunosenescence which may hinder effective viral clearance and lead to a more dysregulated host response. These findings underscore that age is a critical prognostic marker that should be prioritized during the initial triage and clinical management of CCHF patients to optimize outcomes in older populations.

Another critical finding of our study is the role of CRP and fibrinogen as independent biochemical markers reflecting the host's systemic involvement. Consistent with previous studies reporting these parameters as indicators of poor prognosis [6, 7, 12, 13], our data validate their status as significant predictors in the context of CCHF. Elevated CRP levels were independently associated with both mortality and disease severity, highlighting the central role of systemic inflammation. In contrast, fibrinogen demonstrated a more specific association; while it was identified as an independent predictor for disease severity, it lacked independent significance for mortality within the multivariate model. This inverse relationship where lower fibrinogen levels indicate an increased risk of a severe clinical course is consistent with the consumption coagulopathy characteristic of CCHF. The absence of independent significance for fibrinogen in predicting mortality suggests that while coagulopathy is a hallmark of clinical deterioration, the final fatal outcome is governed more decisively by the synergistic effects of viral burden and hyperinflammation.

This finding is further corroborated by the clinical observation in our study that 12 patients initially classified as “mild” based on their admission SGS scores ultimately resulted in mortality. This underscores the highly dynamic and unpredictable clinical progression of CCHF. It suggests that a “mild” status at admission especially when accompanied by a high admission viral load does not necessarily guarantee a favorable prognosis. In such cases, the disease can rapidly transition from a stable phase to terminal multi‐organ failure or massive hemorrhage before the clinical severity scores can reflect the imminent danger. Therefore, admission viral load should be considered a critical early warning sign, even in patients who appear clinically stable at the time of presentation.

Despite the large cohort and comprehensive virological data, this study has limitations. The retrospective design precluded the analysis of dynamic changes in parameters over time. Being a single‐center study, generalizability to different geographical regions or genotypes may be limited. Furthermore, variations in the time from symptom onset to admission may have influenced viral load measurements.

## 5 Conclusion

This study demonstrates that a high admission viral load is the most powerful independent predictor of mortality in CCHF, while host‐related markers specifically CRP and fibrinogen along with patient age, are more decisive for predicting clinical severity. Viral load initiates the fatal outcome as a primary trigger; however, clinical progression is governed by the systemic inflammation and coagulopathy it induces. The combined evaluation of admission viral load, CRP levels, and age provides the most reliable strategy for early risk stratification and the targeted clinical management of high‐risk CCHF patients in clinical practice.

## Author Contributions

**Yasemin Çakır Kıymaz:** conceptualization and writing – original draft, methodology, formal analysis, **Ertuğrul Keskin:** data curation. **Caner Öksüz:** visualization. **Seyit Ali Büyüktuna:** writing – review and editing, investigation. **Nazif Elaldı:** supervision and project administration.

## Funding

The authors have nothing to report.

## Ethics Statement

This study was approved by the Sivas Cumhuriyet University Social Sciences and Humanities Ethics Committee (Decision no: 2025‐09/05, Date: 18.09.2025) and was conducted in accordance with the principles of the Declaration of Helsinki.

## Conflicts of Interest

The authors declare no conflicts of interest.

## Data Availability

The data that support the findings of this study are available on request from the corresponding author. The data are not publicly available due to privacy or ethical restrictions. The authors declare that the data is available.
